# Theoretical procedures of the cross-cultural adaptation process of the Child Mania Rating Scale - Parent Version (CMRS-P) for the Brazilian context

**DOI:** 10.47626/2237-6089-2021-0390

**Published:** 2023-10-31

**Authors:** Tharso de Souza Meyer, Vera Lúcia Marques de Figueiredo, Eric A. Youngstrom, Taiane de Azevedo Cardoso, Jaciana Marlova Gonçalves Araújo, Thaise Mondin, Flávia de Lima Osório, Ilana Andretta, Luciano Dias de Mattos Souza

**Affiliations:** 1 Programa de Pós-Graduação em Saúde e Comportamento Universidade Católica de Pelotas Pelotas RS Brazil Programa de Pós-Graduação em Saúde e Comportamento, Universidade Católica de Pelotas (UCPel), Pelotas, RS, Brazil.; 2 Departments of Psychology and Neuroscience, and Psychiatry University of North Carolina at Chapel Hill Chapel Hill NC USA Departments of Psychology and Neuroscience, and Psychiatry, University of North Carolina at Chapel Hill, Chapel Hill, NC, USA.; 3 Department of Psychiatry and Behavioural Neurosciences McMaster University Hamilton ON Canada Department of Psychiatry and Behavioural Neurosciences, McMaster University, Hamilton, ON, Canada.; 4 Universidade Federal do Rio Grande Rio Grande RS Brazil Universidade Federal do Rio Grande (FURG), Rio Grande, RS, Brazil.; 5 Universidade Federal de Pelotas Pelotas RS Brazil Universidade Federal de Pelotas (UFPel), Pelotas, RS, Brazil.; 6 Departamento de Neurociências e Ciências do Comportamento Faculdade de Medicina de Ribeirão Preto Universidade de São Paulo Ribeirão Preto SP Brazil Departamento de Neurociências e Ciências do Comportamento, Faculdade de Medicina de Ribeirão Preto, Universidade de São Paulo (USP), Ribeirão Preto, SP, Brazil.; 7 Centro de Ciências da Saúde Universidade do Vale do Rio dos Sinos São Leopoldo RS Brazil Centro de Ciências da Saúde, Universidade do Vale do Rio dos Sinos (UNISINOS), São Leopoldo, RS, Brazil.

**Keywords:** Bipolar disorder, mania, children, adolescents, psychiatric status rating scales

## Abstract

**Objectives:**

To describe the theoretical procedures employed in the process of cross-cultural adaptation (CCA) for Brazil of the Child Mania Rating Scale - Parent Version (CMRS-P).

**Methods:**

Seven steps were carried out: (1) translations and synthesis; (2) Committee of Judges-I; (3) grammatical review; (4) Committee of Judges-II; (5) semantic analysis (pre-test); (6) back-translation; and (7) discussion with the authors of the original instrument. Participants were two professional translators, 14 experts, a grammar proofreader, and 21 parents/guardians, representatives of the target population. The results were analyzed in terms of the percentage of agreement between evaluators and the content validity coefficient (CVC) and by analysis of comments and suggestions.

**Results:**

Grammatical and cultural adjustments were made, in addition to substitution and/or inclusion of words and examples. Adequacy agreement indexes exceeding 86% were achieved and the CVC result for the total scale was excellent (0.95). The pre-test indicated good acceptance and understanding by participants.

**Conclusion:**

The proposed version proved to be promising for use in the Brazilian context, although further psychometric studies are still needed to prove the scale’s validity and reliability.

## Introduction

Although bipolar spectrum disorders (BD) with onset in childhood or adolescence have significant negative impact,^1,2^ they are often underdiagnosed and, consequently, undertreated.^3^ BD in children and adolescents is likely to have worse prognosis, including a strong tendency for the onset of clinical and psychiatric comorbidities, in addition to other negative outcomes.^1,2^

Several instruments have been developed internationally for assessment of (hypo)mania in children and adolescents.^1,2^ Currently, it is known that instruments that ask the questions to parents/guardians represent the best choice.^4^ The Child Mania Rating Scale - Parent Version (CMRS-P) stands out for being the first scale developed especially for children and adolescents.^5^ It is based on the Diagnostic and Statistical Manual of Mental Disorders, 4th edition (DSM-IV) criteria and it also includes specific items that concern the main symptoms of BD in children and adolescents.^5^ It consists of a list of behaviors and parents are asked to identify how often these behaviors have occurred with their children in the past month. The CMRS-P is a one-dimensional instrument to be answered in 10-15 minutes.^5,6^ It comprises 21 items assessing frequency of each behavior on a four-point Likert response scale. The results of the original study indicated good psychometric characteristics: internal consistency (α = 0.96), temporal stability (1 week; *r* = 0.96), and validity based on external criteria, in addition to analyses of diagnostic efficiency.^5,6^ The scale also proved its sensitivity to symptomatic changes that happen throughout pharmacological treatment for BD in children and adolescents.^7^ Although it is not a diagnostic tool, it is relevant for differential diagnosis in the assessment of symptoms and even during therapeutic follow-up and evaluation of change in response to treatment.^4-8^

In the Brazilian setting, to date, there are no publications reporting on the cross-cultural adaptation or construction of instruments for assessment of BD in children/adolescents.^9^ Some experts recommend the CMRS-P for assessment of children/adolescents.^1-4^ However, although some initial efforts to provide instruments for assessing BD in children/adolescents have been identified, there is still a gap in the field.

Considering that BD are common and often first manifest in childhood and adolescence, it is important to have assessments to detect (hypo)manic symptoms for this specific developmental phase. Comprehensive studies of cross-cultural adaptation of instruments from other contexts/countries are needed.^9-11^ Thus, the objective of this study is to present the theoretical procedures employed in the process of cross-cultural adaptation (CCA) for the Brazilian context of the CMRS-P scale.

## Methods

**Figure 1:**
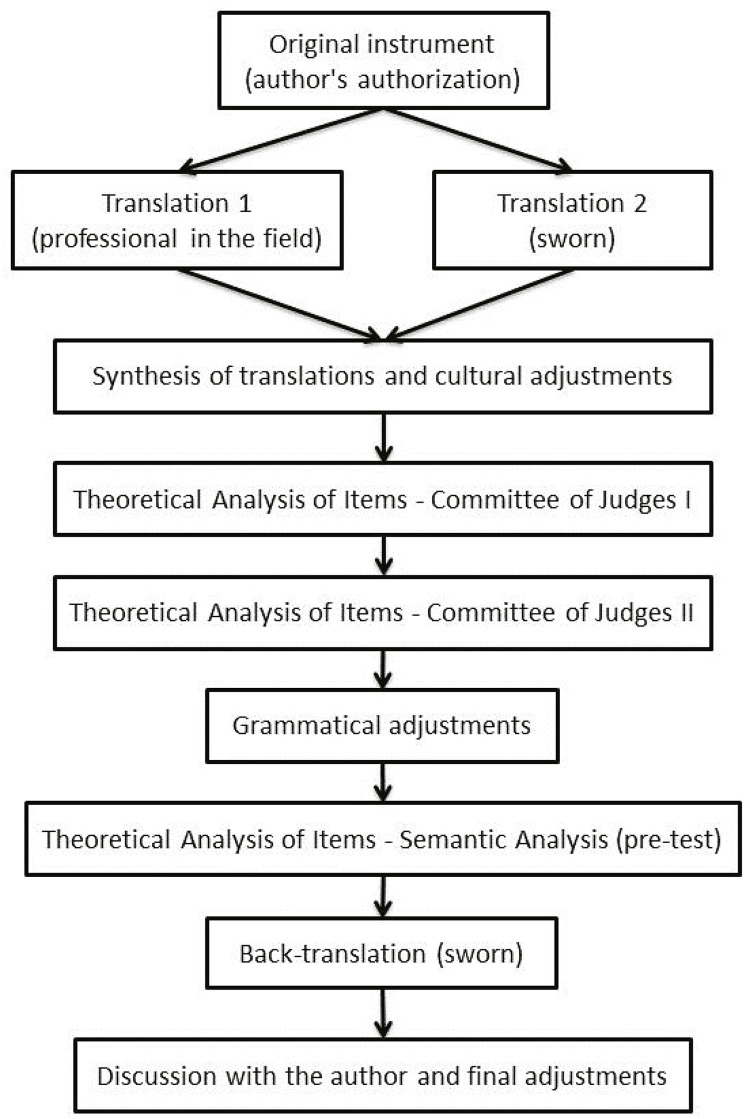
Flowchart illustrating theoretical procedures used in the cross-cultural adaptation process (CCA).

This study addresses the CCA of psychometric instruments,^9-11^ following the three procedures model (theoretical, empirical, and analytical procedures) proposed by Pasquali et al.^10^ This paper describes the theoretical component, consisting of seven stages (Figure 1). The project was approved by the Research Ethics Committee (no. 3,453,369) and authorized by the authors of the original instrument.

The process started with two translations of the original scale, one performed by a bilingual specialist and the other by a sworn translator. The study authors performed the synthesis stage, working with the four versions shown in Table S1 (available as online-only supplementary material), which comprised the original instrument (in English), the two translated versions, and a preliminary version that was used in a clinical pharmacological trial by another research group.^8^ That version was the result of a translation and back-translation process conducted for use in a clinical pharmacological trial. Psychometric analyses were not carried out and no semantic adaptation procedures were described.^8^ The author of that proposal also contributed to the theoretical analysis of the items in the present study.

The resulting version of this stage was forwarded to a Committee of Judges, composed of seven specialists and/or researchers in the fields of psychometry, BD, and/or experts in child/adolescent mental health. The members of this group were sent a form providing general information about the study, a description of the construct evaluated (diagnostic criteria for a manic episode),^2^ instructions and scale items, and space for their qualitative and quantitative assessments of the clarity and adequacy/relevance of the items. At this stage, the judges were blinded to the original instrument.

After making the adjustments suggested by the first Committee, the interim version was sent to a specialist in Portuguese for grammatical correction. It was then considered appropriate to resort to a second Committee of Judges, composed of seven specialists in BD in childhood/adolescence (with studies published in the area) and two professionals in child and adolescent mental health working at two Psychosocial Care Centers for Children and Adolescents (Centro de Atenção Psicossocial Infanto-Juvenil [CAPSi]).

For the pre-test, a convenience sample was selected, consisting of 21 parents/guardians of children/adolescents, who were mostly educated up to high school level (n = 15). The objectives were to verify the comprehensibility, acceptability, and affective impact of the instructions and items with representative members of the target population and to investigate possible linguistic and operational adaptations.

Adjustments were made after the pre-test and the resulting version was sent to another independent sworn translator, blinded to the original instrument, to perform a back-translation. The back-translated version was sent to the authors of the original instrument for quantitative and qualitative assessment.

The analyses performed in this study were primarily qualitative based on the diagnostic criteria of the Diagnostic and Statistical Manual of Mental Disorders, 5th edition (DSM-5).^2^ In the quantitative analyses, the percentage of agreement between evaluators was investigated, considering limits of ^3^ 80% for agreement and ^3^ 0.8 for the content validity coefficient (CVC).

## Results and discussion

The synthesis version was based on the two translations produced for the present study and another version that had been used in a previous study.^8^ The following adjustments were made to the final version, adapted to the Brazilian context: (a) the period of the original scale was maintained – “last month”; (b) exclusion of a sentence from the instructions – referring to abnormal difficulty for the child’s age; (c) inclusion of the option to indicate male or female sex; (d) simplification of some expressions; (e) inclusion of examples; (f) exclusion of repeated words in the same item; and (g) cultural adjustments.

During the analysis of item adequacy for the synthesis version, only item 19 (“Do you have any weird or suspicious thoughts?”) did not reach the pre-established minimum percentage of agreement in the first Committee (Table 1). The first Committee’s review indicated some concerns about the clarity of some items, reaching a minimum agreement threshold for only nine items. Revisions were made based on the feedback from this stage, as follows: (a) extra information on how to fill out the scale was provided; (b) additional emphasis was put on the period under investigation in the statements; (c) additional information and examples were included in the items; (d) expressions considered inappropriate or inefficient were deleted. After these adjustments, the version was sent to a Portuguese language reviewer who recommended some corrections related to verbal tense and phrasal organization and substitution and/or addition of certain words.

**Table 1:** Results of Judge Committees I and II

Items	CJ-I (n = 7)		CJ-II (n = 8)		CVC	

	CL (%)	AD (%)	CL (%)	AD (%)	CL	AD
Instructions	*	*	**57**	86	0.76	0.95
01	71	86	86	100	0.95	1.0
02	86	100	86	100	0.95	1.0
03	57	100	71	86	0.9	0.95
04	**29**	100	**43**	86	0.71	0.95
05	86	100	71	86	0.86	0.95
06	71	100	71	86	0.9	0.95
07	71	100	86	100	0.95	1.0
08	86	100	100	100	1.0	1.0
09	86	100	100	100	1.0	1.0
10	71	100	86	100	0.9	1.0
11	71	100	100	100	1.0	1.0
12	71	100	100	100	1.0	1.0
13	71	100	100	100	1.0	1.0
14	**43**	100	86	100	0.95	1.0
15	86	100	71	100	0.9	1.0
16	100	100	100	100	1.0	1.0
17	100	86	86	100	0.95	1.0
18	71	100	86	100	0.9	1.0
19	**43**	**71**	71	100	0.81	1.0
20	86	86	100	100	1.0	1.0
21	86	86	100	100	1.0	1.0
Pe						0.0000012
CVC_t_						0.95

AD = adequacy/pertinent; CJ-I = Committee of Judges-I; CJ-II = Committee of Judges-II; CL = clarity; CVC = content validity coefficient; CVCt = content validity coefficient of the overall scale; Pe = error calculation.

Bold font indicates lower than expected results.

* The instructions were not assessed quantitatively by the Committee of Judges-I.

Minor adjustments were suggested by the second Committee. The instruction and all items showed more than 80% adequacy agreement (Table 1). Regarding clarity, seven items still showed indexes below expectations. However, the CVC results were also satisfactory (0.95) for the full scale. Besides specific adjustments, the most significant change was inclusion of the expression “Does your child ...” at the beginning of each item of the scale, to help comprehension.

Some of the judges’ questions were about the use of the word “normal”, suggesting the use of a synonym such as usual, habitual, expected, “expected for the age”. However, we decided to retain the word, considering that the term refers to “normal” for a particular case – and not to “normal” for a given population.

In general, the scale was well accepted when presented to the pre-test participants. According to some mothers, the terms used in the items are similar to the language used in written communications produced by schools or other assistance services. Among the main contributions of this group, we highlight substitution of some words and expressions and inclusion of examples. After making this set of adjustments, the version was back-translated and forwarded to the authors of the original version.

Some judges recommended exclusion of some items (17, 19, 20, and 21). However, we believe that in future psychometric analyses the results for each item will justify keeping or excluding them from the scale based on quantitative criteria. One judge recommended greater balance between the items explored and another judge recommended formulation of additional items to assess irritable mood, elevated self-esteem, and hypersexuality. However, it was decided to keep the original form of the scale to fit future cross-cultural psychometric studies, considering that items 2, 3, 4, and 13 assess the specific symptoms emphasized by judges.

As a final result, after the back-translation step, the authors of the original scale, together with other researchers, rated all items and instructions of the scale as “appropriate”. No changes were suggested by the authors.

The objectives related to the theoretical procedures of the CCA of the CMRS-P scale were achieved. The Brazilian version of CMRS-P demonstrated adequate semantic equivalence parameters, enabling its use to investigate mania symptoms in child and adolescent population.
