# Supplementary material for: Theoretical procedures of the cross-cultural adaptation process of the Child Mania Rating Scale - Parent Version (CMRS-P) for the Brazilian context
Source: Trends Psychiatry Psychother. 2023 Oct 31;45:e20210390. doi: 10.47626/2237-6089-2021-0390 (PMC10640885; doi:10.47626/2237-6089-2021-0390)
Supplement: Supplementary file 1 [file 2238-0019-trends-45-e20210390-suppl.pdf]

Table S1 - Child Mania Rating Scale - Parent Version (CMRS-P)

Original	Group UFRGS	Synthesis version I	Synthesis version II	Final version
The following questions concern your child's mood and behavior in the **past month**. Please place a check mark or an 'x' in a box for each item. Please consider it a problem if it is **causing trouble** and is beyond what is normal for your child's age. Otherwise, check 'rare or never' if the behavior is not causing trouble.	As perguntas a seguir referem-se ao humor e ao comportamento do seu filho durante a última semana. Por favor, faça uma marca ou um "x" em um quadrado para cada item. Por favor, considere o item um problema se ele está causando dificuldade e está além do que é normal para a idade do seu filho. Por exemplo, marque "raramente ou nunca" se o comportamento não está causando dificuldade. Períodos significam intervalos de no mínimo 4 horas.	As questões a seguir se referem ao humor e ao comportamento do(a) seu(sua) filho(a) no **último mês**. Por favor, faça um "x" em um quadrado para cada item. Por favor, considere um problema se isso está causando dificuldades e está além do que é considerado normal para a idade do(a) seu(sua) filho(a). Caso contrário, marque "raro ou nunca" se o comportamento não causou problemas no **último mês**.	As questões abaixo se referem ao humor e comportamento do/a seu/sua filho/a no **último mês**. Para respondê-las, faça um 'X' em um quadrado para cada item. Marque em "Raro ou nunca" se o comportamento não causou problemas. Entretanto, se o humor ou comportamento tenha causado dificuldades, estando além do que é considerado normal (ou esperado) para a idade, marque em "Às vezes", "Frequentemente" ou "Muito Frequentemente", conforme a quantidade de vezes que eles ocorreram no **último mês**.	As questões abaixo se referem ao humor e comportamento do/a seu/sua filho/a no **último mês**. Para respondê-las, faça um 'X' em um quadrado para cada item. Marque em "Raro ou nunca" se o comportamento não causou problemas. Entretanto, se o humor ou comportamento tenha causado dificuldades, estando além do que é normal, marque em "Às vezes", "Frequentemente" ou "Muito Frequentemente", conforme a quantidade de vezes que eles ocorreram no **último mês**.
**Does your child...**	**Seu filho...**	**Seu/Sua filho(a)....**	**No último mês, seu/sua filho/a...**	
1. Have periods of feeling super happy for hours or days at a time, extremely wound up and excited, such as feeling "on top of the world"	1. Tem períodos nos quais se sente super feliz por horas ou dias seguidos, extremamente ligado e excitado, como se sentisse "no topo do mundo"	1. Tem períodos que se sente muito feliz por horas ou dias seguidos, extremamente animado(a) e empolgado(a), como se sentindo "no topo do mundo"	1. Teve períodos que se sentiu muito feliz por horas ou dias seguidos (a maior parte do tempo), extremamente animado/a e empolgado/a, como se sentindo "no topo do mundo" com uma alegria exagerada	1. No último mês, seu/sua filho/a teve momentos que se sentiu muito feliz por horas ou dias seguidos (a maior parte do tempo), extremamente animado/a e empolgado/a, como se sentisse "ganhador/a na loteria", com uma alegria exagerada
2. Feel irritable, cranky, or mad for hours or days at a time	2. Sente-se irritável, mal-humorado ou bravo por horas ou dias seguidos	2. Sente-se irritado(a), ranzinza ou furioso(a) por horas ou dias seguidos	2. Sentiu-se irritado/a, ranzinza, rabugento ou furioso/a por horas ou dias seguidos	2. No último mês, seu/sua filho/a sentiu-se irritado/a, ranzinza, rabugento/a ou furioso/a por horas ou dias seguidos
3. Think that he or she can be anything or do anything (e.g., leader, best basket ball player, rap singer, millionaire, princess) beyond what is usual for that age	3. Pensa que pode ser ou fazer qualquer coisa (por exemplo, ser líder, o melhor jogador de futebol, um cantor, um milionário, ou uma princesa) além do normal para a idade	3. Pensa que pode ser ou fazer qualquer coisa (por exemplo, ser líder, o melhor jogador(a) de futebol ou cantor(a), milionário(a), príncipe ou princesa) além do que é normal para a idade	3. Pensou que pode ser ou fazer qualquer coisa (por exemplo, ser grande líder, o melhor jogador/a de futebol ou cantor/a, milionário/a, príncipe/princesa), fora do contexto de brincadeira, além do que é normal para a idade	3. No último mês, seu/sua filho/a pensou que poderia ser ou fazer qualquer coisa (por exemplo, ser um/uma grande líder, o melhor jogador/a de futebol ou cantor/a, milionário/a, príncipe/princesa), fora de um contexto de brincadeira

4. Believe that he or she has unrealistic abilities or powers that are unusual, and may try to act upon them, which causes trouble	4. Acredita que tem habilidades não reais ou poderes incomuns, podendo tentar agir através deles causando problemas	4. Acredita que tem habilidades irreais ou poderes incomuns, e pode tentar agir de acordo as habilidades/poderes, o que causa problemas	4. Teve problemas por acreditar que tem habilidades irreais ou poderes incomuns, agindo de acordo com isso	4. No último mês, seu/sua filho/a teve problemas por acreditar que tinha habilidades que na verdade não tinha, ou superpoderes, agindo de acordo com isso
5. Need less sleep than usual; yet does not feel tired the next day	5. Precisa dormir menos que o normal; não se sente cansado no outro dia	5. Precisa dormir menos que o normal, não se sentindo cansado(a) no dia seguinte	5. Precisou dormir menos que o normal, não se sentindo cansado/a no dia seguinte	5. No último mês, seu/sua filho/a precisou dormir menos que o normal, não se sentindo cansado/a no dia seguinte
6. Have periods of too much energy	6. Tem períodos de energia em demasia	6. Tem períodos de muita energia	6. Teve períodos de muita energia	6. No último mês, seu/sua filho/a teve momentos de muita energia
7. Have periods when she or he talks too much or too loud or talks a mile-a-minute	7. Tem períodos nos quais fala em demasia, muito alto ou fala muito rápido	7. Tem períodos em que fala muito ou muito alto ou muito rápido	7. Teve períodos em que falava muito, ou muito alto, ou muito rápido	7. No último mês, seu/sua filho/a teve momentos em que falava muito, ou muito alto, ou muito rápido
8. Have periods of racing thoughts that his or her mind cannot slow down , and it seems that your child's mouth cannot keep up with his or her mind	8. Tem períodos de pensamentos acelerados, nos quais sua mente não consegue diminuir a velocidade, parecendo que sua boca não consegue acompanhar sua mente	8. Tem períodos de pensamentos acelerados, nos quais sua mente não consegue desacelerar, parecendo que sua fala não consegue acompanhar seus pensamentos	8. Teve períodos de pensamentos acelerados parecendo que sua fala não consegue acompanhar seus pensamentos	8. No último mês, seu/sua filho/a teve momentos de pensamentos acelerados, parecendo que sua fala não conseguia acompanhar seus pensamentos (fala atropelada; como se vomitasse palavras)
9. Talk so fast that he or she jumps from topic to topic	9. Fala tão rápido que pula de assunto em assunto	9. Fala tão rápido que pula de um assunto para outro	9. Falou tão rápido que pulava de um assunto para outro	9. No último mês, seu/sua filho/a falou tão rápido que pulava de um assunto para outro
10. Rush around doing things nonstop	10. Corre por ai, fazendo coisas sem parar	10. Corre por aí fazendo coisas sem parar (estar "a mil por hora")	10. Correu por aí fazendo coisas sem parar (estava "a mil por hora")	10. No último mês, seu/sua filho/a correu por aí fazendo coisas sem parar (estava "a mil por hora")
11. Have trouble staying on track and is easily drawn to what is happening around him or her	11. Tem dificuldade para manter-se focado e é facilmente distraído pelo que está acontecendo ao seu redor	11. Tem dificuldade para manter-se focado(a) e é facilmente distraído(a) pelo que está acontecendo ao seu redor	11. Teve dificuldade para manter-se focado/a, sendo facilmente distraído/a pelo que está acontecendo ao seu redor	11. No último mês, seu/sua filho/a teve dificuldade para manter-se atento/a, sendo facilmente distraído/a pelo que estava acontecendo ao seu redor
12. Do many more things than usual, or is unusually productive or highly creative	12. Faz muito mais coisas que o normal; é produtivo mais do que o normal ou altamente criativo	12. Faz muito mais coisas do que o habitual, está mais produtivo(a) do que o normal ou altamente criativo(a)	12. Fez muito mais coisas do que o habitual, estava mais produtivo/a ou altamente criativo/a	12. No último mês, seu/sua filho/a fez muito mais coisas do que o normal, estava mais produtivo/a ou altamente criativo/a (fazendo muitas coisas novas)

13. Behave in a sexually inappropriate way (e.g., talks dirty, exposing, playing with private parts, masturbating, making sex phone calls, humping on dogs, playing sex games, touches others sexually)	13. Age de forma sexualmente inapropriada (por exemplo, conversa suja, exibindo ou brincando com as partes íntimas, masturbando-se, fazendo telefonemas eróticos, curvando-se sobre cachorros, brincando com jogos sexuais, toca os outros sexualmente)	13. Comporta-se de uma forma sexualmente inadequada (por exemplo, fala obscena, exibe ou brinca com partes íntimas, se masturba, busca por conteúdos sexuais, imita sexo com animais, toca os outros sexualmente)	13. Comportou-se de uma forma sexualmente inadequada (por exemplo, falou palavras obscenas, exibiu ou brincou com partes íntimas, se masturbou, buscou por conteúdos sexuais na internet, imitou sexo com animais ou tocou os outros sexualmente)	13. No último mês, seu/sua filho/a comportou-se de uma forma sexualmente inadequada (por exemplo, falou palavras obscenas/palavrão, exibiu ou brincou com as próprias partes íntimas, se masturbou, buscou por conteúdos sexuais na internet, imitou sexo com animais ou tocou outras pessoas sexualmente)
14. Go and talk to strangers inappropriately, is more socially outgoing than usual	14. Vai e fala com estranhos de forma inapropriada; é mais saliente socialmente do que o normal	14. Fala com estranhos de forma inadequada, é mais socialmente extrovertido(a) do que o normal	14. Falou ou agiu com estranhos de forma inadequada, sendo mais socialmente extrovertido/a do que o normal (mais expansivo, descontraído, desinibido)	14. No último mês, seu/sua filho/a falou ou agiu com estranhos de forma inadequada, sendo mais extrovertido/a do que o normal (mais expansivo, descontraído, desinibido)
15. Do things that are unusual for him or her that are foolish or risky (e.g., jumping off heights, ordering CDs with your credit cards, giving things away)	15. Faz coisas que não são usuais para ele, que são arriscadas ou insensatas (por exemplo, pular de alturas, encomendar CDs com os seus cartões de crédito, jogar pertences fora)	15. Faz coisas incomuns para ele(a) que sejam tolas ou arriscadas (por exemplo, pular de alturas, fazer compras com seu dinheiro sem sua permissão, doar coisas importantes)	15. Fez coisas incomuns para ele/a que foram tolas ou arriscadas (por exemplo, pulou de alturas, fez compras com dinheiro dos outros sem permissão, doou coisas importantes sem permissão)	15. No último mês, seu/sua filho/a fez coisas incomuns para ele/a que foram tolas ou arriscadas (por exemplo, pulou de alturas, fez compras com dinheiro dos outros ou doou coisas importantes sem permissão)
16. Have rage attacks, intense and prolonged temper tantrums	16. Tem ataques de fúria ou crises de birra intensas e prolongadas	16. Tem ataques de fúria ou crises de birra intensas e prolongadas?	16. Teve ataques de fúria ou crises de birra intensas e prolongadas?	16. No último mês, seu/sua filho/a teve ataques de fúria ou crises de birra intensas e prolongadas?
17. Crack jokes or pun more than usual, laugh loud, or act silly in a way that is out of the ordinary	17. Conta piadas ou faz trocadilhos mais do que o normal, ri alto ou age de maneira boba de uma forma que é fora do seu usual	17. Faz piadas ou brincadeiras mais do que o normal, ri alto ou age de maneira boba, fora do comum?	17. Fez mais piadas ou brincadeiras do que o normal, riu alto demais ou agiu de maneira boba, fora do comum?	17. No último mês, seu/sua filho/a fez mais piadas ou brincadeiras do que o normal, riu alto demais ou agiu de maneira boba, fora do comum?
18. Experience rapid mood swings	18. Tem flutuações rápidas de humor	18. Experimenta mudanças rápidas de humor?	18. Teve mudanças rápidas de humor?	18. No último mês, seu/sua filho/a teve mudanças rápidas de humor?
19. Have any suspicious or strange thoughts	19. Tem algum pensamento estranho ou de desconfiança	19. Tem algum pensamento estranho ou de desconfiança?	19. Teve algum pensamento estranho (bizarro, fora do contexto que ele/a está) ou de desconfiança?	19. No último mês, seu/sua filho/a teve algum pensamento estranho (bizarro, fora do contexto que ele/a estava) ou de desconfiança?
20. Hear voices that nobody else can hear	20. Ouve vozes que mais ninguém consegue ouvir	20. Ouve vozes que ninguém mais pode ouvir?	20. Ouviu vozes que ninguém mais pode ouvir?	20. No último mês, seu/sua filho/a ouviu vozes ou barulhos que ninguém mais podia ouvir?
21. See things that nobody else can see	21. Vê coisas que mais ninguém consegue ver	21. Vê coisas que ninguém mais pode ver?	21. Viu coisas que ninguém mais pode ver?	21. No último mês, seu/sua filho/a viu coisas que ninguém mais podia ver?
